# Factors associated with the rabies vaccination status of dogs in households in Beni City, D.R. Congo

**DOI:** 10.4102/ojvr.v91i2.2168

**Published:** 2024-08-16

**Authors:** Prince D. Kimpanga, Emery K. Taghembwa, Gauthier M. Mubenga, Jean-Marie T. Makwera, Norbert M. Muhongya, Odrade B. Chabikuli, Justin M. Masumu

**Affiliations:** 1 Department of Epidemiology and Biostatistics, Faculty of Medicine, University of Kinshasa, Kinshasa, Democratic Republic of the Congo; 2 Department of Veterinary Inspectorate, North Kivu Provincial Bureau of Animal Production and Health, Beni, Democratic Republic of the Congo; 3 African Field Epidemiology Network (AFENET). Kinshasa, Democratic Republic of the Congo; 4 Higher Institute of Medical Techniques, Beni, Democratic Republic of the Congo; 5 Faculty of Medicine, Université Officielle de Ruwenzori, Butembo, Democratic Republic of the Congo; 6 Faculty of Veterinary Medicine, Université Nationale Pédagogique, Kinshasa, Democratic Republic of the Congo

**Keywords:** rabies, vaccination, dog, zoonosis, household survey, Beni

## Abstract

Human rabies transmitted by dogs still kills thousands of people each year worldwide. Dog bites are common in the city of Beni (Democratic Republic of Congo), which shows low rabies vaccination coverage. This study aimed to determine the factors associated with the rabies vaccination status of dogs. A cross-sectional analytical study was conducted in the town of Beni among dog owners, during a household survey selected using a multistage sampling. The information sought concerned the knowledge and characteristics of the dog owners as well as the vaccination status of these dogs. Logistic regression was used to investigate associations between the vaccination status of the dogs and the main independent factors. Rabies vaccination coverage in Beni was 26% (95% confidence interval [CI]: 22% – 30%). The main factors associated with the rabies vaccination status of the dog were primary education level of household head (adjusted odds ratio [aOR]:4.8; 95% CI: 1.2– 19.8); university education level of household head (aOR: 5.9; 95% CI: 1.6–22); perceived rabies severity (aOR: 44. 4; 95% CI: 10.4–188), having more than one dog in the household (aOR: 2.6; 95% CI: 1.6–4.3); age range 7–12 months (aOR: 0.2; 95% CI: 0.1–0.6) and confined dog breeding (aOR: 3.9; 95% CI: 1.1–14.9). The low vaccination coverage in Beni requires mass vaccination campaigns against canine rabies targeting the dog owners with low education levels, those raising more than one dog, with stray dogs or dogs less than 12 months old.

## Introduction

Rabies is a zooanthroponosis that causes a viral encephalomyelitis common to many animal species and humans, with fatal outcomes. Although a preventable disease, the number of rabies cases in developing countries is high. This number is usually under-reported (Talbi et al. 2009). In most developing country settings, passive rabies surveillance appears to be ineffective and community-based active surveillance has shown its limitations (Kitala et al. 2000). Dog-transmitted human rabies still kills tens of thousands of people worldwide a year. The number of human deaths because of dog-transmitted rabies is estimated at 59 000 per year worldwide (World Health Organization [WHO] 2021).

The Global Framework for Rabies Elimination was launched in 2015 with a common global goal of zero human deaths from dog-transmitted rabies by 2030. In endemic countries, the WHO recommends the immediate initiation of post-exposure prophylaxis (PEP) after a bite from a rabies suspect animal (Hampson et al. 2015). Given the cost of PEP and its limited access in low-income countries (Medley et al. 2017), the central pillar of dog-transmitted rabies control is large-scale dog vaccination campaigns (WHO 2021). This requires a one-health approach based on close collaboration and partnership between the human, animal and environmental health sectors but with the involvement and commitment of local populations.

**FIGURE 1:**
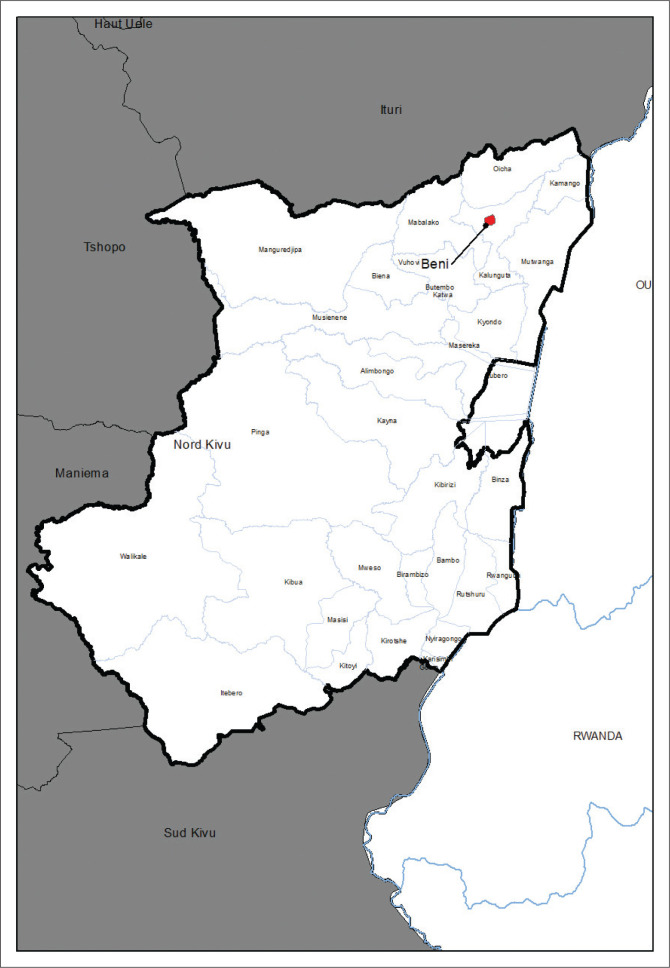
Beni City located in the North-East of the Democratic Republic of the Congo and close to the Virunga National Park and the country’s border with Uganda.

In the Democratic Republic of the Congo (DRC), data from the City of Kinshasa showed a reduction in the frequency of rabies cases between 2009 and 2011 followed by a recent increase in cases number until 2013 (Twabela et al. 2016). According to routine surveillance data reported in Kinshasa, 21 human deaths out of 3726 cases of suspicious bites were recorded between 2016 and 2021 (Office of Vaccination Against Rabies and Central Kinshasa veterinary laboratory, 2021), while 157 human deaths were recorded out of 10 498 cases of suspicious bites between 2010 and 2020 in Katanga (Vaccination Office of Lubumbashi, Haut Katanga province, 2020). Of the 143 samples taken and tested in dogs at the National Biomedical Research Institute (INRB) between 2017 and 2019, 93 were positive for rabies (PANLIRA-RDC 2021). Located in the north-east of the DRC and close to the Virunga National Park and the country’s border with Uganda (Figure 1), the town of Beni records a high frequency of people being bitten by dogs and most of these victims do not access the PEP. The Provincial Bureau of Animal Production and Health reported low vaccination coverage against dog rabies (23%) in 2012. The reasons for this low rabies vaccination coverage in Beni are still unclear.

This study would like to answer the question why this low rabies vaccination coverage? The aim of this study is to determine the factors associated with the rabies vaccination status of dogs in the town of Beni.

## Research methods and design

### Setting

**FIGURE 2:**
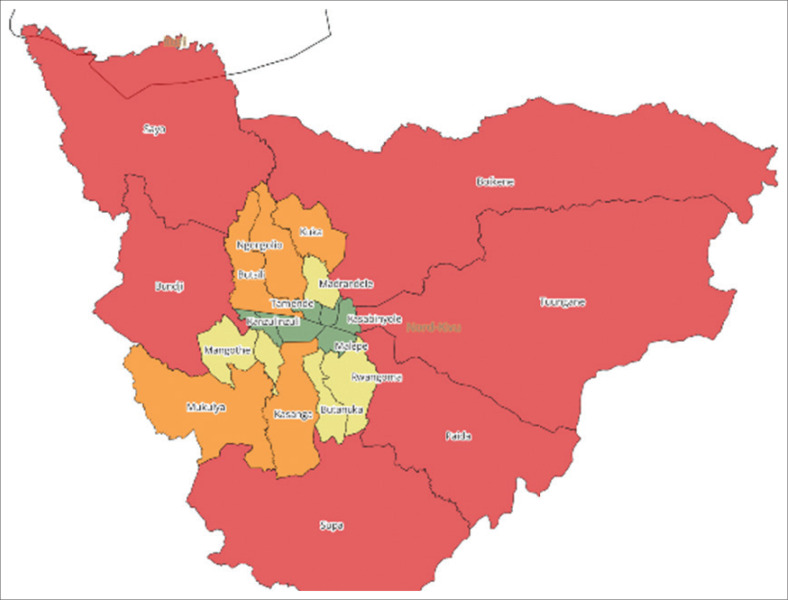
The health areas in the health zone of Beni.

This study was conducted throughout the town of Beni, which corresponds to the urban health zone of Beni. It has a population of 474 236 inhabitants spread over a geographical area with an average density of 386 inhabitants/km^2^. This health zone is divided into 18 health areas (HAs) (Figure 2) and includes 306 healthcare structures, including 1 general reference hospital, 3 hospital centres and 18 health centres.

### Design and study population

A cross-sectional analytical study was conducted between November 2021 and February 2022, among dog owners in a household survey in the Beni Health Zone. This study targeted dog owners living in households in HAs in the town of Beni. These were people who own at least one dog at least 3 months old during the study period.

### Sampling

The Epi Info sample size calculator was used to determine the 500 households based on some hypotheses (18% expected frequency, 5% acceptable margin of error [MOE], 2 design effects and 10% of non-responders). The study participants (dog owners in the households) were selected using a multistage sampling. The 18 HAs in the town were included in two separate lists: a list of 12 urban HAs and another list of 6 rural HAs. In the first stage, 6 HAs included in the study were randomly selected: 4 from the list of 12 urban HAs and 2 from the list of 6 rural HAs. At the second stage, 30 avenues were randomly selected from the 6 HAs included in the study, with 5 avenues for each of the HAs selected. A list of plots or households owning at least one dog was conducted for each avenue selected for the study. In the third stage, a random selection of 510 households was made from the list of households included in the plot list of each avenue. As 30 avenues were selected for the study, the selection of households was made based on 17 households per avenue.

### Data collection

Trained interviewers collected data. They conducted face-to-face interviews with the dog owners using a pre-tested questionnaire. This was structured questionnaire containing a mixture of closed and open questions, written in French and translated into the local language (Swahili). The information sought concerned the characteristics and vaccination status of the dogs, the socio-demographic characteristics and the knowledge of the owners of the dogs found in the households.

### Data analysis

The data for this study were entered into the computer using Epi data 3.1 software, and the data analysis was performed with SPSS^®^ version 27.0. Quantitative variables were summarised by calculating means and standard deviations while qualitative variables are presented in the frequency tables with their confidence intervals. The dependent variable ‘vaccination status’ was categorised into two: vaccinated versus non-vaccinated. For multiple-dog households, vaccination status was considered ‘vaccinated’ if at least one dog in the household was vaccinated. Associations were sought between the dog’s vaccination status and the independent factors. Firstly in bivariate analysis using the Chi-square test of independence at a significance level α = 0.05 and the odds ratio (OR) with its 95% confidence interval (95% CI). When an association was suspected (*p*-value ≤ 0.20) in bivariate analysis, the factor concerned was entered into a logistic regression model that identified the factors associated with the dog’s rabies vaccination status.

## Results

The sample selected for this study included a total of 500 households with at least one dog. Households in the urban part of the Beni city represented 74% of the sample. About a third of these households had more than one dog. In general, dogs were less aggressive (59%) and were bred (88%). Only 12% of respondents admitted that their dogs were stray. Most of the dogs (81%) were used for reasons other than hunting (guarding or pets). The participants in this household survey were predominantly male (69%) living with a partner (83%), with a secondary (34%) or university (35%) education level and employed (75%).

Of the 500 households surveyed, 26% reported that their dogs had received the rabies vaccine in the previous year. This vaccination coverage is within a 95% CI of 22% – 30%. Most households with vaccinated dogs reported having received their rabies vaccine at the official veterinary service (83%). Forty-two per cent of households usually visit the veterinary service (95% CI: 38% – 46%).

The bivariate analysis between the dog’s vaccination status and the socio-demographic characteristics of the owner and household head (Table 1) shows that vaccination status is not associated with religion, nor with the size of the household, nor with the fact that the head of household has a job or not. On the other hand, the vaccinated dogs were more frequent in urban areas, in the presence of a male head of household, living with a partner, and the chance of having a vaccinated dog increases with the educational level of the owner (household head).

**TABLE 1:** Dog vaccination status and socio-demographic characteristics of the household.

Socio-demographic characteristics	Vaccinated (*n* = 130)		Non-vaccinated (*n* = 370)		cOR	95% CI	*p*-value
	*n*	%	*n*	%			
**Setting**							
Rural	21	16.2	111	30.0	1.00	-	-
Urban	109	83.8	259	70.0	2.22	1.33-3.73	0.002
**Gender**							
Female	28	21.5	126	34.1	1.00	-	-
Male	102	78.5	244	65.9	1.88	1.18-3.01	0.008
**Marital status**							
Living alone	12	9.2	75	20.3	1.00	-	-
Living with a patner	118	90.8	295	79.7	2.50	1.31-4.77	0.005
**Religion**							
Revival	19	14.6	74	20.0	1.00	-	-
Catholic	63	48.5	165	44.6	1.49	0.83-2.66	0.181
Protestant	48	36.9	131	35.4	1.43	0.78-2.61	0.248
**Education level**							
None	3	2.3	59	15.9	1.00	-	-
Primary	16	12.3	79	21.4	3.98	1.11-14.3	0.034
Secondary	41	31.5	134	36.2	6.02	1.79-20.2	0.004
University	70	53.8	98	26.5	14.10	4.23-46.6	0.000
**Job**							
No	36	27.7	87	23.5	1.00	-	-
Yes	94	72.3	283	76.5	0.80	0.51-1.26	0.324
**Household size**							
> 6 persons	95	73.1	254	68.6	1.00	-	-
≤ 6 persons	35	26.9	116	31.4	0.81	0.52-1.26	0.345

cOR, crude odds ratio; CI, confidence interval.

Most respondents have heard of rabies. But although knowledge is low on the transmission (12%) and prevention (24%) of rabies, the vaccinated dogs were associated with some of the respondent’s knowledge on rabies (Table 2): having heard of rabies, knowledge of mode of transmission, the severity of rabies, prevention and number of vaccine doses required per year.

**TABLE 2:** Dog vaccination status and respondents’ knowledge on rabies.

Respondents’ knowledge	Vaccinated (*n* = 130)		Non-vaccinated (*n* = 370)		cOR	95% CI	*p*-value
	*n*	%	*n*	%			
**Have heard of rabies**							
No	1	0.8	30	8.1	1.00	-	-
Yes	129	99.2	340	91.9	11.40	1.54-84.3	0.017
**Transmission mode**							
No	122	93.8	319	86.2	1.00	-	-
Yes	8	6.2	51	13.8	0.41	0.19-0.89	0.024
**Rabies severity**							
No	2	1.5	171	46.2	1.00	-	-
Yes	128	98.5	199	53.8	55.00	13.4-225.6	0.000
**Clinical signs**							
No	21	16.2	78	21.1	1.00	-	-
Yes	109	83.8	292	78.9	1.39	0.82-2.36	0.227
**Rabies prevention**							
No	80	61.5	301	81.4	1.00	-	-
Yes	50	38.5	69	18.6	2.73	1.76-4.23	0.000
**Number of doses per**							
No	3	2.3	37	10.0	1.00	-	-
Yes	127	97.7	333	90.0	4.70	1.43-15.5	0.011

cOR, crude odds ratio; CI, confidence interval.

The vaccinated dogs were associated with certain animal characteristics (Table 3): the ownership of more than one dog, the non-aggressive behaviour, guard or companion dog (non-hunting use) and dog bred in confinement (no straying dog).

**TABLE 3:** Dog vaccination status and animals’ profile in the household.

Animals profile	Vaccinated (*n* = 130)		Non-vaccinated (*n* = 370)		cOR	95% CI	*p*-value
	*n*	%	*n*	%			
**Household dog number**							
One dog	73	56.2	266	71.9	1.00	-	-
More than one	57	43.8	104	28.1	1.99	1.32-3.02	0.001
**Sex**							
Male	85	65.4	206	55.7	1.00	-	-
Female	45	34.6	164	44.3	0.67	0.44-1.01	0.054
**Age (in months)**							
3 to 6	19	14.6	50	13.5	1.00	-	-
7 to 12	14	10.8	73	19.7	0.51	0.23-1.10	0.085
More than 12	97	74.6	247	66.8	1.03	0.58-1.84	0.911
**Aggressive behavior**							
Yes	77	59.2	128	34.6	1.00	-	-
No	53	40.8	242	65.4	0.36	0.24-0.55	0.000
**Hunting dog**							
Yes	6	4.6	87	23.5	1.00	-	-
No	124	95.4	283	76.5	6.35	2.70-14.9	0.000
**Stray dog**							
Yes	3	2.3	59	15.9	1.00	-	-
No	127	97.7	311	84.1	8.03	2.47-26.1	0.001

cOR, crude odds ratio; CI, confidence interval.

Logistic regression identified the most important factors associated with the dog’s rabies vaccination status (Table 4). The vaccinated dogs were more frequent in households headed by people who have been to school compared to those headed by people with no education. The vaccinated dogs were observed five and six times more in households whose head has completed primary school (*p* < 0.05) and university education (*p* < 0.01), respectively. The vaccinated dogs were 44 times more observed when the household head perceives the rabies severity (*p* < 0.001). Vaccinated dogs were twice as high in households with more than one dog (*p* < 0.001) compared to households with only one dog.

**TABLE 4:** Multivariate logistic regression assessing the association between dog vaccination and independent factors.

Veterinary services	Vaccinated (*n* = 130)		Non-vaccinated (*n* = 370)		cOR	95% CI	aOR	95% CI	*p*-value
	*n*	%	*n*	%					
**Education level**									
None	3	2.3	59	15.9	1.00	-	-	-	-
Primary	16	12.3	79	21.4	3.98	1.11-14.3	4.77	1.15-19.8	0.031
Secondary	41	31.5	134	36.2	6.02	1.79-20.2	3.45	0.91-13.1	0.069
University	70	53.8	98	26.5	14.10	4.23-46.6	5.89	1.57-22.1	0.009
**Rabies severity**									
No	2	1.5	171	46.2	1.00	-	-	-	-
Yes	128	98.5	199	53.8	55.00	13.4-225	44.4	10.4-188	0.000
**Household dog numbers**									
One dog	73	56.2	266	71.9	1.00	-	-	-	-
More than one	57	43.8	104	28.1	1.99	1.32-3.02	2.60	1.57-4.30	0.000
**Dog age (months)**									
3 to 6	19	14.6	50	13.5	1.00	-	-	-	-
7 to 12	14	10.8	73	19.7	0.51	0.23-1.10	0.24	0.09-0.63	0.004
More than 12	97	74.6	247	66.8	1.03	0.58-1.84	0.55	0.26-1.15	0.114
**Aggressive behavior**									
Yes	77	59.2	128	34.6	1.00	-	-	-	-
No	53	40.8	242	65.4	0.36	0.24-0.55	0.65	0.40-1.06	0.084
**Stray dog**									
Yes	3	2.3	59	15.9	1.00	-	-	-	-
No	127	97.7	311	84.1	8.03	2.47-26.1	3.99	1.07-14.9	0.040

cOR, crude odds ratio; aOR, adjusted odds ratio; CI, confidence interval.

Certain dog characteristics were also associated with rabies vaccination status. The vaccinated dogs were four times less frequent in households with dogs aged between 7 months and 12 months (*p* < 0.01) compared to those aged between 3 months and 6 months. Finally, the vaccinated dogs were four times more observed in households with dogs kept in confinement (*p* < 0.05) compared to households with dogs said to be strays.

## Discussion

This study aimed to determine the factors associated with the rabies vaccination status of dogs in the town of Beni. The study results show a rabies vaccination coverage of 26% of dog-owning households. Twelve per cent of the households owned stray dogs. Respondents with secondary or university education dominated. They had heard of rabies, but their knowledge of rabies was low. The most important factors associated with the rabies vaccination status of the dog were: the education level of the household head, his perception of the rabies severity, the number of dogs in the household and the dog’s profile such as age and breeding in confinement.

The rabies vaccination coverage measured in this study is lower than that observed in a commune of Kinshasa (49%) by the team of Kazadi et al. (2017). The methodological differences between the two studies may explain the discrepancy between these two results. In the ‘owner-paid’ scheme, the vaccination coverage observed in Beni is higher than the average canine rabies vaccination coverage of the eleven African countries (18%); compared to the ‘free vaccination’ scheme, the vaccination coverage observed in Beni is much lower than the average of the eleven African countries (68%) (Jibat, Hogeveen & Mourits 2015). In the DRC, rabies vaccination of dogs is usually paid for by the owner. The ‘free vaccination’ scheme, usually subsidised, is possible during outbreaks. To eliminate or prevent rabies outbreaks in a given population, the WHO recommends that 70% of dogs be vaccinated. Based on the estimates of Coleman and Dye (1996), the rabies vaccination coverage observed in Beni is far from the critical percentage (39% – 57%) to justify reaching the 70% required to eliminate or prevent rabies outbreaks.

In the ‘the owner-paid’ dog rabies vaccination scheme, there are factors that may explain low vaccination coverage. The dog’s vaccination status may be associated with the socio-demographic characteristics of the owner, the owner’s knowledge about rabies (Ndenge et al. 2021) and the characteristics of the dogs. According to the results of this study, the vaccinated dogs were more observed in the households where the dog owners have a high level of education. The education level of the dog owner may facilitate the understanding of rabies, its mode of transmission, its prevention and improve its adherence and commitment to rabies vaccination. This result is similar to that obtained by the team of Kazadi et al. (2017) who additionally considered the effect of the cost of vaccination, which our team could not estimate.

According to this study’s results, the number of vaccinated dogs was more when the household head was familiar with the rabies severity. The motivation to vaccinate the dog may reasonably result from the perception of rabies severity. This perception may be influenced by various factors such as breed of dog, which were not studied here. The vaccinated dogs were twice as observed in households with more than one dog compared with households with one dog. The number of dogs in the household is a motivating factor for dog rabies vaccination. Breeding of several dogs requires much more investment from the owner. As the high number of dogs in a household may be an indicative of the socio-economic status of the owner, it suggests important factors that may facilitate the dog’s rabies vaccination.

Among dog characteristics, the vaccinated dogs were less frequent in households with dogs between 7 months and 12 months of age. Dog owners are accustomed to vaccinating dogs according to their age, and the privilege is given to the oldest dogs (12 months and older). They believe that from the age of 12 months onwards, the mature dog becomes defensive and dangerous and may bite people.

The vaccinated dogs were more observed in households with dogs kept in confinement compared to households with dogs that are said to be strays. Keeping dogs in confinement requires a great deal of attention and care from the animal’s owner. Rabies vaccination is an important part of the care of a confined dog. On the other hand, stray dogs are left to own devices and are found in street corners, around markets, in rubbish bins looking for food to satisfy their needs. In such an environment stray dogs are victims to a variety of ailments of different natures, and in order to defend themselves, sometimes inflict bites on their human aggressors.

## Conclusion and perspectives

Rabies vaccination coverage for dogs is low in the town of Beni. It is far from the 70% threshold required by the WHO to eliminate or prevent rabies outbreaks. The most important factors associated with the rabies vaccination status of the dog are the high level of education of the household head, his or her perception of rabies severity, the number of dogs in the household and the dog’s profile such as age and confinement breeding.

The low proportion of vaccinated dogs combined with the proximity of Beni city to the wildlife of Virunga Park and to the Ugandan border of the DRC indicates a need to strengthen cross-border rabies surveillance. In order to ensure collective immunity in the canine population, mass vaccination campaigns must be organised after training the veterinary agents involved in canine rabies vaccination in Beni city. These campaigns should primarily target the dog owners with low education level, those who keep a lot of dogs, those with stray dogs and with dogs less than 12 months old, in order to guarantee collective immunity in the population.
